# Atlas and Epitome of Diseases Caused by Accidents

**Published:** 1901-03

**Authors:** 


Atlas and Epitome of Diseases Caused by Accidents, by Dr. Ed. Golebiewski, of Berlin. Authorized Translation from the German with Editorial Notes and Additions by Pearce Bailey, M. D., Consulting Neurologist to St. Luke's Hospital New York; Assistant in Neurology, Columbia University; Author of "Accidents and Injuries; Their Relation to Diseases of the Nervous System." 40 colored plates, and 143 illustrations in black; pp. 548; price, $4.00. Philadelphia: W. B. Saunders & Co., 1900.

These atlases are intended to serve the purpose of clinical material, but they are poor substitutes. The present volume, though abounding in colored plates, gives the student a poor idea in many instances of the accident sustained. For instance Plate II shows the picture of a man covered with skin, with moustache, hair on head and pubis. From the various colors one might suppose the patient had jaundice or had been pickled for dissection. There is absolutely nothing else striking in the picture, but it is intended to show a "direct fracture of the eighth, ninth and tenth ribs near the vertebral column, and of indirect fracture of the seventh and eighth ribs, or of their cartilage, in the mamilary line, complicated by fracture of the body of the ninth or tenth vertebra."

Much useless work in a number of the plates has been put on the penis and testicles, to give them an artistic appearance, when the object is to show an accident below the knees. But there are many of the plates that are good and the text is clear and valuable.

